# Transient Photocurrent Response of Plasmon-Enhanced Polymer Solar Cells with Gold Nanoparticles

**DOI:** 10.3390/ma8074050

**Published:** 2015-07-06

**Authors:** Yi Fang, Yanbing Hou, Yufeng Hu, Feng Teng

**Affiliations:** 1 Key Laboratory of Luminescence and Optical Information, Ministry of Education, Institute of Optoelectronic Technology, Beijing Jiaotong University, Beijing 100044, China; E-Mails: fangyi@bigc.edu.cn (Y.F.); yfhu@bjtu.eud.cn (Y.H.); fteng@bjtu.eud.cn (F.T.); 2 School of Printing and Packaging Engineering, Beijing Institute of Graphic Communication, Beijing 102600, China

**Keywords:** polymer solar cells, gold nanoparticles, plasmon, transient photocurrent

## Abstract

In this work, the transient photocurrent of the plasmon-enhanced polymer bulk heterojunction solar cells based on poly(3-hexylthiophene) (P3HT) and [6,6]-Phenyl C_61_ butyric acid methyl ester (PCBM) is investigated. Two kinds of localized surface plasmon resonance (LSPR) enhanced devices were fabricated by doping the gold nanoparticles (Au NPs) into the anode buffer layer and inserting Au NPs between the anode buffer layer and the active layer. We probed the dynamics of the turn-on and turn-off responses to 400 μs square-pulse optical excitation from the 380 nm and 520 nm light-emitting diodes (LED) driven by an electric pulse generator. The transient photocurrent curves of devices with Au NPs at different positions and under different excitation wavelength are compared and analyzed. The charge trapping/detrapping processes that occurred at the interface of Au NPs and the active layer were observed; these exhibit an overshoot in the initial fast rise of photocurrent response. Our results show that the incorporating position of Au NPs is an important key factor to influence the transient photocurrent behaviors.

## 1. Introduction

Polymer solar cells (PSCs) have attracted broad interests as a kind of clean and renewable energy source, due to their unique advantages, such as light weight, low cost, low-temperature fabrication, and compatibility with flexible substrates [1,2]. The power conversion efficiency (PCE) of PSCs based on tandem structure has reached 10.6% and the single junction architecture has also already reached an efficiency of 8.6% [3,4]. However, the efficiency of PSC is still low compared with commercial products. One of the main reasons for this is attributed to the fact that broad band cannot provide an appropriate overlap with the solar spectrum, which limits the sunlight absorption of the active layer [5]. Some promising approaches, such as developing novel low-band-gap polymer semiconductors, inserting optical spacers into the cells and designing new device architectures, have been proposed in many literatures [6,7,8,9,10]. Besides the mentioned approaches, localized surface plasmon resonance (LSPR) is also an attractive approach in which metallic nanoparticles are incorporated into the devices, due to its potential for concentrating and channeling light in the active layer [11,12,13,14]. The LSPR effect of Ag or Au nanoparticles has been successfully utilized in various photovoltaic devices to yield plasmon-enhanced photocurrents and enhance their efficiencies [15,16,17,18,19,20,21,22,23,24]. 

Charge transport needs to overcome the barrier between the active layer and the electrode or buffer layer in PSCs [25]. The defect-induced local accumulation of charge and the increasing charge density which hamper the internal electric field in the devices may result in recombining or trapping separated charges, leading to the space-charge effect [26]. Despite the fact that the role that the embedded metallic nanoparticles play in charge separation and transport is an interesting issue, the literature on this topic is limited. On the one hand, the metallic nanoparticles can improve the conductivity of the region where they are located (reduce the series resistance of the device); on the other hand, the metallic nanoparticles may lead to the charge carrier quenching or recombination due to the residual surfactant or surface defects around the nanoparticles. These processes occurred in a very short time; thereby, the steady state photocurrent measurement cannot provide detailed information.

Transient photocurrent measurements can provide direct information about the dynamics of the charge transport and charge trapping/detrapping process. Here we investigate the Au NPs plasmon-enhanced effect in PSCs through the measurement of transient short-circuit current, which can help us to understand the enhancement effect of Au NPs on the performance of PSCs. The results show that both the LSPR effect and the scattering effect tracing to the Au NPs can increase the generation of charge carriers. However, the Au NPs enhanced photocurrent is reduced by their trapping effect of charge carriers.

## 2. Experimental

**Figure 1:**
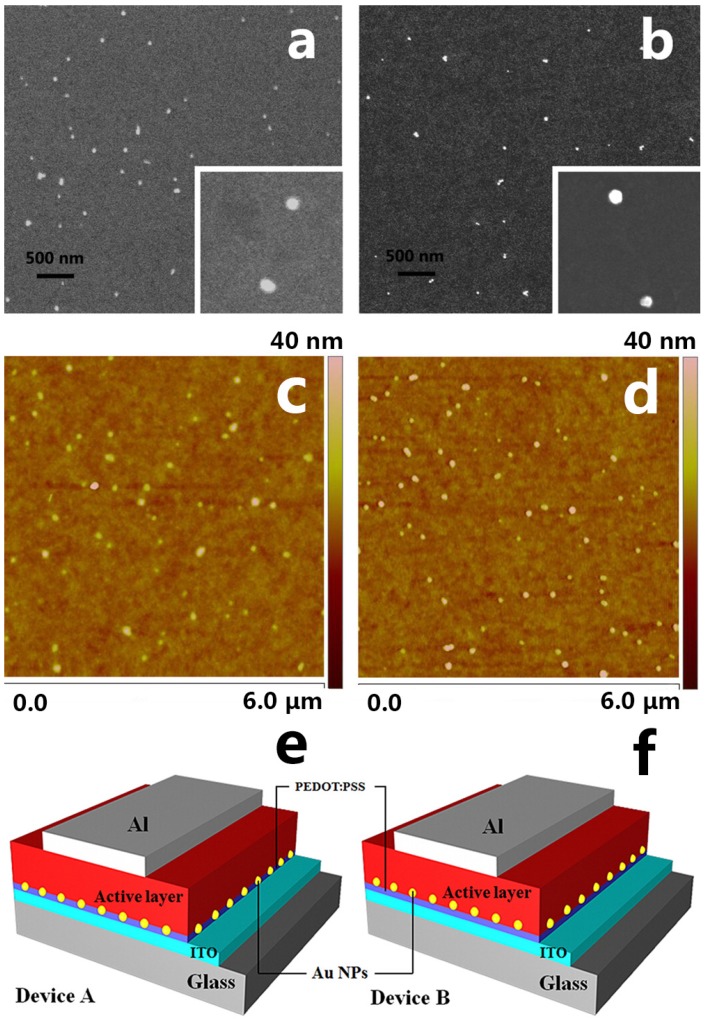
Scanning electron microscopy (SEM) images of gold nanoparticles (Au NPs) blend into poly(3,4-ethylenedioxythiophene) poly(styrenesulfonate) (PEDOT:PSS) film (**a**) and on the PEDOT:PSS film (**b**); atomic force microscope (AFM) images of Au NPs blend into PEDOT:PSS film (**c**) and on the PEDOT:PSS film (**d**); the structures of two plasmon-enhanced devices (**e**,**f**).

The colloidal synthesis of Au NPs was reported [27,28]. The fabricated Au NPs have an average diameter of 50 nm. The presence of Au NPs incorporated with the poly(3,4-ethylenedioxythiophene) poly(styrenesulfonate) (PEDOT:PSS) layer and inserted between the PEDOT:PSS layer and the active layer was proved by the scanning electron microscopy (SEM) images shown in Figure 1a,b. The distribution of Au NPs in the modified PEDOT:PSS layer and above on the PEDOT:PSS layer are uniform. There is no apparent aggregation of Au NPs in or on PEDOT:PSS films. Figure 1c,d shows the corresponding images captured by atomic force microscope (AFM). The light spots represent the Au NPs and their height (the lighter the color is, the greater the height of the Au NPs is). The average height of the Au NPs in Figure 1d is about 15 nm greater than the average height of the Au NPs in Figure 1c by analyzing the AFM images. The result indicates that the positions of the Au NPs by two methods are different.

The structures of the experimental devices are ITO/PEDOT:PSS:Au NPs/P3HT:PCBM/Al (device A) and ITO/PEDOT:PSS/AuNPs/P3HT:PCBM/Al (device B). For the sake of comparison, a control device (device C) without Au NPs was also fabricated under the same conditions. Figure 1e,f displays the structures of device A and B. ITO-coated glass substrates were first cleaned in boiled deionized water with detergent, then ultrasonicated in acetone, and eventually dried with nitrogen gas. The PEDOT:PSS layer of device A was spin-cast onto the pre-cleaned substrates from water-based PEDOT:PSS solution (Baytron P, 4083, HC Stark) blended with 0.5 wt % Au NPs colloid. Then PEDOT:PSS layers were annealed at 150 °C for 20 min. For device B, Au NPs colloid dispersed in ethanol solution with a concentration of 0.5 wt % was spin-cast onto the annealed pristine PEDOT:PSS layer. In order to eliminate the residual ethanol, the substrates with Au NPs were annealed again at 150 °C for 10 min. Then, the dichlorobenzene solution consisting of P3HT (Rieke Metals Inc) and PCBM (Nichem Fine Technology Co. Ltd., Jhubei, Taiwan) was spin-cast. The concentrations of both P3HT and PCBM are 20 mg/mL. Lastly, to complete the devices, a 100 nm thick Al film was deposited by thermal evaporation under a vacuum of 5 × 10^−6^ Torr. The active area of the devices is 9 mm^2^.

The transient photocurrent measurement was performed under the illumination of 380 nm and 520 nm LEDs driven by a pulse generator (AVTECH, AV-1011-B). The duration and frequency were 400 μs and 50 Hz respectively. The rise time and fall time of the light pulse are less than 0.5 μs. Photocurrent was measured by connecting the device to a digitizing oscilloscope (TEK, TDS540D) with the input impedance of 50 Ω. The devices were kept in the dark until the measurement was carried out. Intensities of the light pulse were adjusted by changing the voltage applied to the LEDs. The external quantum efficiency (EQE) of the devices was measured with Zolix Solar Cell Scan 100 (Zolix Instrument Co. Ltd., Beijing, China). Under the illumination of AM1.5G 100 mW/cm^2^, the current density-voltage (*J*-*V*) characteristics were performed with a Keithley 2410 sourcemeter under ambient conditions.

## 3. Discussion

**Figure 2:**
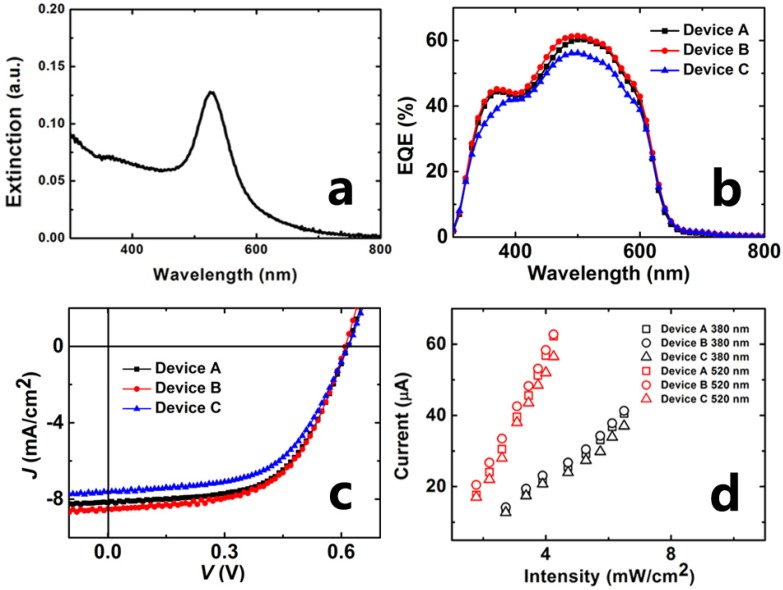
The extinction spectrum of the colloidal solution of Au NPs (**a**); the external quantum efficiency (EQE) curves (**b**); *J*-*V* characteristics of devices under AM1.5G 100 mW/cm^2^ illumination (**c**); the steady-state short-circuit current of devices under the illumination of 380 nm light and 520 nm light with various intensities (**d**).

Figure 2a shows the extinction spectrum of the Au NPs recorded by the UV-Vis Scanning Spectrophotometer (UV-3101PC, SHIMADZU Co. Ltd., Kyoto, Japan), which exhibits a distinct extinction peak at 550 nm. The EQE spectra (Figure 2b) reveal that the EQE responses of device A and B are significantly enhanced by the incorporation of Au NPs, comparing with device C without Au NPs. The enhancements of the EQE of device A and B appear in two wavelength regions: the short wavelength region and the plasmon resonance region. In the case of the devices with Au NPs, the enhanced EQE in 450–600 nm are attributed to the plasmonic effect easily [29], while the enhancements of EQE in the short wavelength region can be attributed to the scattering effect of the Au NPs. Our previous work has recorded the extinction spectrum of Au NPs film (with the UV-Vis Scanning Spectrophotometer) and the absorption curve of the film of Au NPs (with an integrating sphere, a xenon lamp and an indicator) [30].

The steady-state photovoltaic characteristics of the plasmon-enhanced devices and the control device are shown in Figure 2c and Table 1. The PCEs of device A and B increase 11% and 12.9% respectively, comparing with the device C. The *J*_SC_ of device A and B increases 7.2% and 11.4%. The increase in PCE is mainly attributed to the enhancement of *J*sc. The steady photocurrent of three devices exhibits a near-linear dependence on the light intensity under the illumination of both 380 nm and 520 nm light as shown in Figure 2d. The *J*_SC_ of three devices *versus* light intensity curves can be fitted by a relation of *J*_SC_ ∝ α*I*, where *I* is the light intensity, and α is a constant. The constant α is more affected by the illumination wavelength and less affected by the devices. The α values of device A, B and C under the illumination of 380 nm light are 6.96, 6.82 and 6.05, respectively. While the α values of device A, B and C under the illumination of 520 nm light are 18.0, 17.2 and 16.4, respectively.

**Table 1:** The steady-state characteristics of devices.

Devices	*J*_SC_ (mA/cm^2^)	*V*_OC_ (V)	Fill factor	PCE (%)
Device A	8.18	0.62	0.58	2.93
Device B	8.50	0.62	0.57	2.98
Device C	7.63	0.62	0.56	2.64

**Figure 3:**
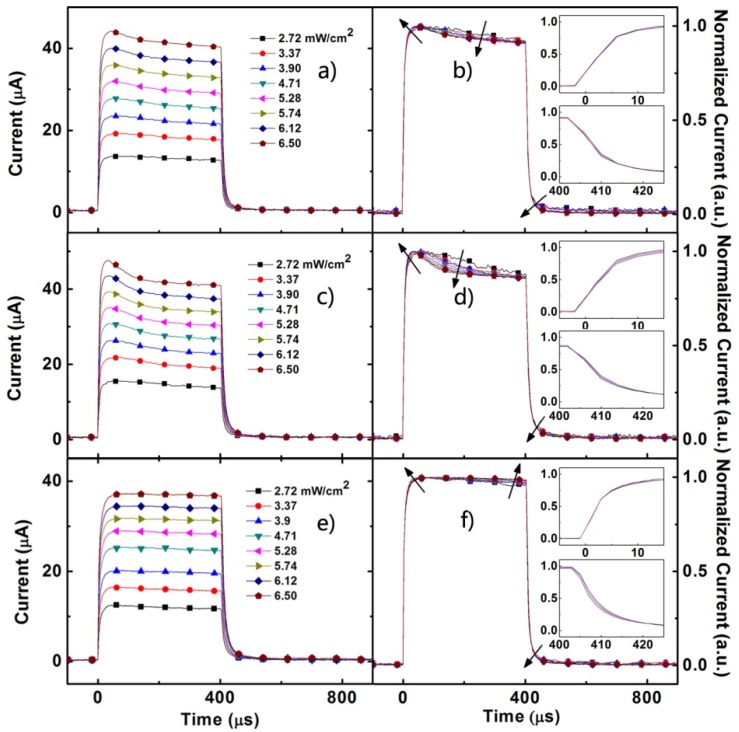
The normal (left) and normalized (right) transient short-circuit photocurrent of device A (**a**,**b**) device B (**c**,**d**) and device C (**e**,**f**) under illumination of 380 nm pulse with various intensities. The insets (**b**,**d**,**f**) show the detail of rise/fall dynamics.

The transient short-circuit current (TSCC) of three devices under the illumination of 380 nm light pulse are presented in Figure 3. For the device without Au NPs, the turn-on and turn-off dynamics of the control device (Figure 3e) seem to be independent of the illumination intensity. However, the normalized current curves (Figure 3f) can present the slight change in short-circuit current response with the increasing intensity of illumination. With increasing light intensity, two changes in the TSCC are found: (1) a systematic decrease in the time taken to reach steady state, which may be consistent with charge density dependence of mobility [31,32]; (2) in the later stage of turn-on state, response shows more flat with the increasing light intensity.

Figure 3a,c shows the TSCC response of the devices with Au NPs under the 380 nm illumination of different light-intensity. The changes in the dynamics with the light intensity are similar as described in some literatures [33,34]. The TSCC responses of both device A and B show a fast initial rise with an overshoot in the turn-on, and the overshoot phenomenon is enhanced with increasing light intensity. Compared with device A, the overshoot phenomenon of device B is more apparent. The overshoot phenomenon reflects the charge accumulation resulting from the non-equilibrium transport of electrons and holes in the devices [33,34]. The charge accumulation would lead to the redistributed electric field, which can cause the decrease in field-dependent exciton separation and carrier transport. When the light is on, the incorporated Au NPs in the front part of the active layers would enhance the light absorption of active layers. Generally, the Au NPs’ enhanced light absorption might result from two causes. First, the scattering effect lengthens the optical path in the active layer, thereby enhancing the light absorption. Alternatively, the excitation of the LSPR resulted in local enhancement of the electromagnetic field in the vicinity of the Au NPs. Then, the plasmon-exciton coupling might facilitate exciton dissociation [21], which can be explained in terms of fast free-carrier transport. Meanwhile, the slower dynamics are associated with the trapping/detrapping processes to reach steady state after the turn-on, or after the turn-off [35]. The Au NPSs not only increase the photo-generated charge carriers, but also introduce the traps which cause the overshoot phenomenon. Compared with the device without Au NPs, the normalized current curves of the devices with Au NPs (Figure 3b,d) show that the overshoot is more apparent with the increase in light intensity, and the time to reach the quasi-steady-state is shortened, which implies the equilibration between trapping and detrapping is reached. Furthermore, the insets in Figure 3b,d exhibit the rise/fall dynamics which evolve to the steady state in several microseconds.

**Figure 4:**
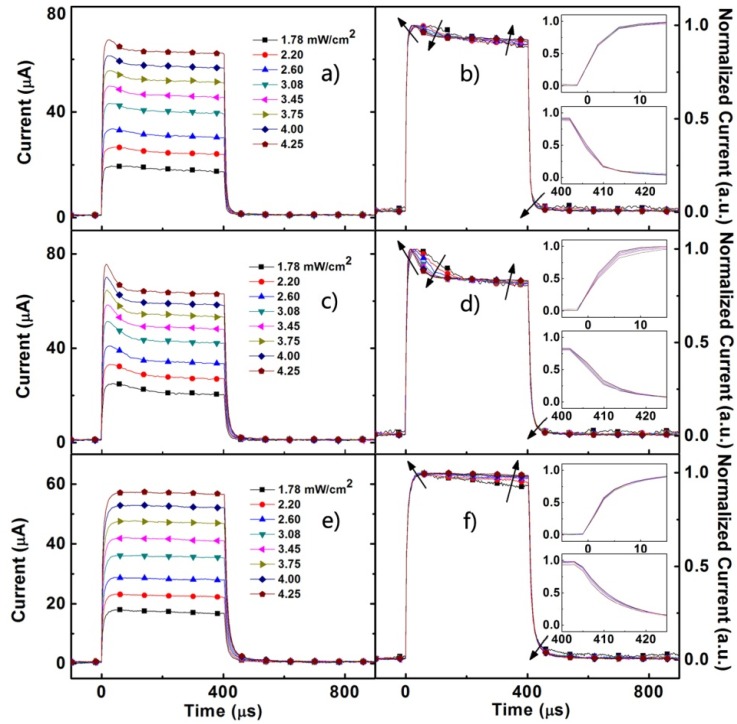
The normal (left) and normalized (right) transient short-circuit photocurrent of device A (**a**,**b**) device B (**c**,**d**) and device C (**e**,**f**) under illumination of 520 nm pulse with various intensities. The insets (**b**,**d**,**f**) show the subtle change in rise/fall dynamics.

Under the excitation of 520 nm, the TSCC dynamics of the control device (Figure 4e,f) is still independent of the light-intensity. The short-circuit photocurrent dynamics of the devices with Au NPs (in particular device B) show the more apparent overshoot in the turn-on of the photocurrent evolved with increasing light intensity (Figure 4a,c), which reveals that the charge trapping processes occurred around the Au NPs due to surface states or defects. A similar phenomenon also occured in the solar cells based on CdSe nanocrystals doped polymer [36,37]. The charges, in particular electrons, may be trapped at the interface between the Au NPs and the active layer without a corresponding detrapping process. The sharper TSCC overshoot in device B indicates that the magnitude of trapped carries becomes larger with the increasing contacted area between the Au NPs and the active layer. The steep overshoot indicates that Au NPs bring a strong trapping process due to larger photocurrent response resulting from the high efficiency at 520 nm. Moreover, the insets in Figure 4b,d also represent the rise/fall dynamics under the illumination of 520 nm light. 

**Figure 5:**
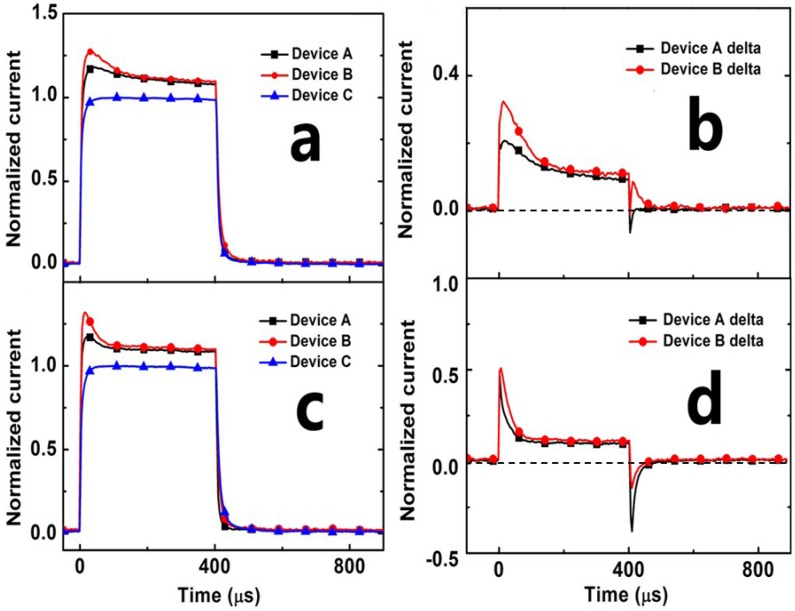
Normalized current response curve comparison of devices A, B and C (**a**,**c**); the normalized Δ*I*_SC_ of devices A and B (**b**,**d**). Both devices were under the maximum intensity of 380 nm (**a**,**b**) and 520 nm (**c**,**d**) light illumination.

Figure 5a,c show the normalized TSCC response of devices under 380 nm and 520 nm light illumination with their maximum intensity. The overshoot of device B is apparently sharper. The declining process of its overshoot is faster than that of device A, which indicates more charge carriers generated with more traps emerged in device B. Although the LSPR effect indeed increases the photo-generated carriers, the photo-generated carriers are hampered by the trapped-charge effects of Au NPs. The Δ*I_SC_* = (TSCC_X_-TSCC_C_)/TSCC_C_ (X:A or B) under 380 nm and 520 nm light illumination with their maximum intensity are presented in Figure 5b,d. 

Because the enhanced EQE at 520 nm comes from LSPR of Au NPs at 520 nm, the more photogenerated carriers are close to Au NPs, and transient overshoot under the illumination of 520 nm is stronger, which suggests that the trapping process occurs at the interface between Au NPs and the P3HT/PCBM blend. 

## 4. Conclusions

In summary, we investigate the transient photocurrent of two kinds of plasmon-enhanced devices. The results reveal that the charge trapping/detrapping processes occur at the interface of Au NPs and the active layer, which results in an overshoot in the initial fast rise of photocurrent response. The trapping/detrapping process induced by Au NPs may happen at metal surface states, defects, or surfactants. The enhanced LSPR effect of Au NPs is partly weakened by the trapping of the carriers at the surface of the Au NPs and the polymer. 
